# Hospital Decision-Making and Adoption of Health-Related Social Needs Programs in US Hospitals

**DOI:** 10.1001/jamanetworkopen.2025.16351

**Published:** 2025-06-17

**Authors:** Dina Zein, Cory E. Cronin, Neeraj Puro, Berkeley Franz, Elizabeth McNeill, Ji E. Chang

**Affiliations:** 1 Department of Public Health Policy and Management, New York University School of Global Public Health, New York, New York; 2 Department of Social and Public Health, Ohio University, Athens; 3 Appalachian Institute to Advance Health Equity Science, Athens, Ohio; 4 Florida Atlantic University College of Business, Boca Raton; 5 Ohio University Heritage College of Osteopathic Medicine, Athens

## Abstract

This cross-sectional study examines associations among hospital management involvement in health equity goals and the hospital’s adoption of social needs initiatives.

## Introduction

In response to health disparities in the US,^1^ the Centers for Medicare & Medicaid Services (CMS) released a Framework for Health Equity recommending increased hospital commitment and leadership engagement around screening for health-related social needs (HRSNs).^2^ Research indicates that committed and engaged health care leadership is necessary to advance health equity.^3^ However, little is known about senior management’s role in shaping hospital efforts to screen for and address unmet social needs to improve health equity. Our study examines the association between hospital management involvement in health equity goals and the hospital’s adoption of social needs initiatives.

## Methods

This cross-sectional study used the 2022 American Hospital Association (AHA) Annual Survey to assess hospital characteristics, including HRSN program participation and management involvement. Hospitals not reporting on HRSN programs and management roles in health equity goals were excluded; weights were constructed to adjust for hospital nonresponse (eMethods 1 in Supplement 1). We linked the data to the community characteristics from the 2020 Agency for Healthcare Research and Quality Social Determinants of Health Database^4^ and the 2020 Area Deprivation Index (ADI).^5^ This study of deidentified hospital-level data was exempt from the New York University institutional review board and informed consent, in accordance with 45 CFR §46. We followed the STROBE reporting guidelines.

Separate multivariable logistic regression analyses focused on the 9 individual HRSNs from the AHA survey and a composite measure of having at least 1 of the 5 common HRSNs prioritized by CMS, examining each program delivery and management involvement. The AHA survey categorized management roles into CEO (chief executive office), designated senior executive, middle management, committee/task force, division/department leaders, and employee resource groups. We created a categorical variable indicating whether only senior management (CEO or designated senior executive), only other management, both, or neither were involved (eMethods 2 in Supplement 1). We used StataSE software version 18.5 (StataCorp) for all statistical analyses. Significance was set at 2-sided *P* < .05 (eMethods 3 in Supplement 1).

## Results

Of 4288 acute-care hospitals, 2271 (53.0%) responded to HRSN program and health equity goals items, with 93.9% offering any of the 5 HRSN programs and 1475 (69.2%) reporting both senior and other management involvement (Table 1). The most commonly offered HRSN programs were health behaviors (86.1%), transportation (83.8%), food insecurity (82.7%), and interpersonal violence (69.6%). The least offered program was employment (52.1%) (Table 1).

**Table 1.** Prevalence of HRSNs by Management Involvement and Hospital Characteristics

Characteristic	Hospitals, No. (%)										
	All observations (N = 2271)	Any 5 HRSNs (n = 2133)^a^	Housing (n = 1537)	Food insecurity or hunger (n = 1878)	Transportation (n = 1903)	Utility needs (n = 1271)	Interpersonal violence (n = 1581)	Health behaviors (n = 1955)	Education (n = 1287)	Employment and income (n = 1182)	Social isolation (n = 1556)
Management involvement											
None	106 (4.7)	83 (3.9)	55 (3.6)	63 (3.4)	69 (3.6)	48 (3.8)	55 (3.5)	82 (4.2)	54 (4.2)	48 (4.1)	53 (3.4)
Senior only	487 (21.4)	447 (21.0)	303 (19.7)	377 (20.1)	395 (20.8)	224 (17.6)	296 (18.7)	389 (19.9)	230 (17.9)	214 (18.1)	299 (19.2)
Senior and others	1536 (67.6)	1475 (69.2)	1113 (72.4)	1341 (71.4)	1331 (69.9)	953 (75.0)	1157 (73.2)	1372 (70.2)	938 (72.9)	877 (74.2)	1137 (73.1)
Others only	142 (6.3)	128 (6.0)	66 (4.3)	97 (5.2)	108 (5.7)	46 (3.6)	73 (4.6)	112 (5.7)	65 (5.1)	43 (3.6)	67 (4.3)
Bed size											
<50	729 (32.1)	648 (30.4)	403 (26.2)	531 (28.3)	532 (28.0)	329 (25.9)	403 (25.5)	592 (30.3)	350 (27.2)	300 (25.4)	451 (29.0)
50-199	756 (33.3)	711 (33.3)	511 (33.2)	622 (33.1)	648 (34.1)	418 (32.9)	531 (33.6)	657 (33.6)	424 (32.9)	402 (34.0)	509 (32.7)
200-399	443 (19.5)	435 (20.4)	341 (22.2)	405 (21.6)	406 (21.3)	282 (22.2)	355 (22.5)	394 (20.2)	274 (21.3)	254 (21.5)	320 (20.6)
≥400	343 (15.1)	339 (15.9)	282 (18.3)	320 (17.0)	317 (16.7)	242 (19.0)	292 (18.5)	312 (16.0)	239 (18.6)	226 (19.1)	276 (17.7)
Ownership type											
Nonprofit	1814 (79.9)	1738 (81.5)	1287 (83.7)	1576 (83.9)	1551 (81.5)	1058 (83.2)	1303 (82.4)	1596 (81.6)	1064 (82.7)	998 (84.4)	1294 (83.2)
Public	372 (16.4)	323 (15.1)	213 (13.9)	254 (13.5)	288 (15.1)	183 (14.4)	231 (14.6)	303 (15.5)	185 (14.4)	156 (13.2)	223 (14.3)
For-profit	85 (3.7)	72 (3.4)	37 (2.4)	48 (2.6)	64 (3.4)	30 (2.4)	47 (3.0)	56 (2.9)	38 (3.0)	28 (2.4)	39 (2.5)
Religious											
No	2001 (88.1)	1876 (88.0)	1345 (87.5)	1651 (87.9)	1679 (88.2)	1127 (88.7)	1406 (88.9)	1742 (89.1)	1144 (88.9)	1049 (88.7)	1386 (89.1)
Yes	270 (11.9)	257 (12.0)	192 (12.5)	227 (12.1)	224 (11.8)	144 (11.3)	175 (11.1)	213 (10.9)	143 (11.1)	133 (11.3)	170 (10.9)
Hospital location											
Rural	431 (19.0)	365 (17.1)	200 (13.0)	283 (15.1)	295 (15.5)	155 (12.2)	205 (13.0)	344 (17.6)	194 (15.1)	146 (12.4)	246 (15.8)
Urban	1840 (81.0)	1768 (82.9)	1337 (87.0)	1595 (84.9)	1608 (84.5)	1116 (87.8)	1376 (87.0)	1611 (82.4)	1093 (84.9)	1036 (87.6)	1310 (84.2)
Safety-net status											
No	1896 (83.5)	1775 (83.2)	1274 (82.9)	1568 (83.5)	1586 (83.3)	1072 (84.3)	1324 (83.7)	1632 (83.5)	1087 (84.5)	990 (83.8)	1310 (84.2)
Yes	375 (16.5)	358 (16.8)	263 (17.1)	310 (16.5)	317 (16.7)	199 (15.7)	257 (16.3)	323 (16.5)	200 (15.5)	192 (16.2)	246 (15.8)

Abbreviation: HRSN, health-related social need.

^a^ Any 5 HRSNs includes housing, food insecurity or hunger, transportation, utility needs, and interpersonal violence.

Five of the 9 HRSN programs were significantly associated with this dual involvement, even after controlling for hospital and community characteristics (Table 2). Hospitals with both management levels engaged had higher odds of offering any of the 5 common HRSN programs (odds ratio [OR], 4.08; 95% CI, 2.19-7.60), as well as food insecurity (OR, 2.99; 95% CI, 1.81-4.96), transportation (OR, 2.34; 95% CI, 1.43-3.84), interpersonal violence (OR, 2.22; 95% CI, 1.40-3.50), social isolation (OR, 2.28; 95% CI, 1.42-3.65), and housing (OR, 1.79; 95% CI, 1.13-2.86) compared with hospitals with no management involvement. Meanwhile, having senior management involvement alone only increased odds of offering food insecurity and transportation programs.

**Table 2.** Adjusted Regression Results for HRSN Programs by Management Involvement

Variable	OR (95% CI)									
	Any 5 HRSN^a^	Housing	Food insecurity or hunger	Transportation	Utility needs	Interpersonal violence	Health behaviors	Education	Employment and income	Social isolation
Management involvement (reference: none)										
Senior only	2.28 (1.18-4.38)^b^	1.32 (0.81-2.17)	1.72 (1.01-2.93)^b^	1.83 (1.08-3.10)^b^	0.86 (0.53-1.40)	1.19 (0.73-1.93)	0.85 (0.46-1.58)	0.67 (0.41-1.09)	0.71 (0.43-1.17)	1.38 (0.84-2.27)
Senior and others	4.08 (2.19-7.60)^c^	1.79 (1.13-2.86)^b^	2.99 (1.81-4.96)^c^	2.34 (1.43-3.84)^c^	1.50 (0.95-2.38)	2.22 (1.40-3.50)^c^	1.62 (0.89-2.95)	1.14 (0.72-1.81)	1.15 (0.72-1.84)	2.28 (1.42-3.65)^c^
Others only	2.32 (0.99-5.42)	0.89 (0.48-1.64)	1.45 (0.74-2.82)	1.79 (0.92-3.47)	0.60 (0.32-1.12)	1.02 (0.57-1.83)	0.99 (0.47-2.10)	0.93 (0.51-1.71)	0.47 (0.25-0.86)^b^	0.85 (0.47-1.54)
Organizational characteristics										
Bed size (reference: <50)										
50-199	0.89 (0.54-1.49)	1.23 (0.93-1.62)	1.15 (0.82-1.62)	1.54 (1.09-2.18)^b^	1.21 (0.93-1.56)	1.37 (1.04-1.82)^b^	1.42 (0.97-2.08)^b^	1.23 (0.95-1.59)	1.32 (1.01-1.71)^b^	1.08 (0.82-1.42)
200-399	2.40 (0.95-6.10)	1.47 (1.05-2.07)^b^	2.02 (1.23-3.30)^c^	2.67 (1.69-4.24)^c^	1.42 (1.03-1.95)^b^	1.86 (1.31-2.62)^c^	1.25 (0.79-1.98)	1.44 (1.06-1.96)^b^	1.31 (0.97-1.78)^b^	1.07 (0.77-1.49)
≥400	3.86 (1.27-11.73)^b^	2.02 (1.37-2.96)^c^	2.86 (1.64-4.99)^c^	2.73 (1.65-4.51)^c^	1.85 (1.30-2.61)^c^	2.40 (1.60-3.61)^c^	1.62 (0.99-2.64)	2.03 (1.45-2.84)^c^	1.87 (1.33-2.62)^c^	1.76 (1.21-2.56)^c^
Ownership (reference: for-profit)										
Nonprofit	3.30 (1.65-6.58)^c^	2.90 (1.77-4.75)^c^	4.78 (2.86-7.99)^c^	1.72 (0.97-3.06)	2.74 (1.67-4.50)^c^	2.07 (1.29-3.31)^c^	4.27 (2.52-7.22)^c^	1.72 (1.08-2.74)^b^	2.58 (1.59-4.19)^c^	2.88 (1.79-4.63)^c^
Public	1.32 (0.64-2.72)	1.94 (1.13-3.30)^b^	1.90 (1.09-3.30)^b^	1.29 (0.70-2.37)	2.19 (1.27-3.75)^c^	1.63 (0.98-2.73)	2.44 (1.38-4.31)^c^	1.35 (0.81-2.24)	1.77 (1.05-3.00)^b^	1.85 (1.10-3.11)^b^
Religious	1.02 (0.54-1.95)	1.03 (0.77-1.39)	0.82 (0.56-1.21)	0.84 (0.58-1.23)	0.82 (0.62-1.08)	0.68 (0.50-0.92)^b^	0.42 (0.30-0.60)^c^	0.77 (0.59-1.00)	0.77 (0.59-1.02)	0.65 (0.49-0.87)^c^
Hospital location (reference: rural)										
Urban	2.09 (1.26-3.47)^c^	1.65 (1.23-2.21)^c^	1.85 (1.30-2.62)^c^	1.58 (1.12-2.22)^c^	1.80 (1.34-2.41)^c^	1.76 (1.32-2.35)^c^	1.20 (0.83-1.72)	1.25 (0.94-1.66)	1.79 (1.34-2.38)^c^	1.25 (0.93-1.69)
Safety net hospital	1.72 (0.87-3.40)	1.07 (0.80-1.44)	1.10 (0.76-1.60)	1.08 (0.74-1.58)	0.84 (0.64-1.11)	0.86 (0.64-1.15)	1.09 (0.73-1.62)	0.81 (0.62-1.05)	1.04 (0.79-1.37)	0.88 (0.66-1.17)
County characteristics										
Percentage Black	1.01 (0.99-1.02)	1.01 (1.00-1.02)^b^	1.00 (0.99-1.01)	1.00 (0.99-1.01)	1.01 (1.00-1.02)^b^	1.01 (1.00-1.02)^b^	1.01 (1.00-1.02)^b^	1.00 (0.99-1.01)	1.00 (0.99-1.01)	1.01 (1.00-1.01)
Percentage Hispanic	1.00 (0.98-1.02)	1.02 (1.01-1.03)^c^	1.01 (0.99-1.02)	1.00 (0.99-1.01)	1.01 (1.00-1.02)	1.01 (1.00-1.02)^c^	1.00 (0.99-1.01)	1.01 (1.00-1.01)	1.00 (1.00-1.01)	1.01 (1.00-1.02)^b^
Percentage uninsured	0.99 (0.95-1.03)	0.99 (0.96-1.01)	0.99 (0.96-1.02)	1.01 (0.98-1.04)	1.00 (0.98-1.02)	1.00 (0.97-1.02)	0.98 (0.95-1.01)	1.01 (0.99-1.03)	0.98 (0.96-1.00)	0.98 (0.96-1.01)
Area Deprivation Index	0.99 (0.98-1.00)	0.99 (0.99-1.00)^b^	1.00 (0.99-1.00)	0.99 (0.99-1.00)^c^	1.00 (0.99-1.00)	0.99 (0.99-1.00)^c^	1.00 (0.99-1.00)	1.00 (1.00-1.00)	1.00 (1.00-1.00)	1.00 (1.00-1.01)

Abbreviations: HRSN, health-related social needs; OR, odds ratio.

^a^ Any 5 HRSN includes housing, food insecurity or hunger, transportation, utility needs, and interpersonal violence.

^b^ *P* < .05.

^c^ *P* < .01.

Other hospital characteristics including larger size (≥400 vs <50 beds), nonprofit status (vs for-profit), and nonreligious status (vs religious) had higher odds of offering some, but not all, HRSN programs. Meanwhile, hospitals in counties with more Black and Hispanic residents had higher odds of offering housing and interpersonal violence programs (Table 2).

## Discussion

This cross-sectional study found that hospitals with multiple layers of management engagement tended to adopt multifaceted strategies that address patients’ social needs, which are critical components of health equity frameworks.^2^ These findings align with research emphasizing engaged hospital leadership in driving systemic change to promote health equity.^4^ Interestingly, hospitals where only senior management was involved were more likely to offer specific programs like food insecurity and transportation services, although these associations were generally smaller compared with when both senior and other management were engaged. This suggests that while senior leadership plays a pivotal role, the inclusion of other managers and other organizational layers may be important to implement and sustain comprehensive social services. This aligns with literature on change management, which emphasizes the need for early involvement of multiple stakeholders to maintain successful organizational changes.^6^ Limitations to this study include the inability to infer causality and a 53.0% survey response rate for HRSN program and health equity items. Future work should focus on understanding the role of management in the implementation of successful programs and strategies.

## Notes

Supplement 1. **eMethods 1.** Survey Weights for Survey Nonresponse **eMethods 2.** Creating Categorical Variables for Management Participation in Health Equity Goals **eMethods 3.** Multivariable Logistic Regression

Supplement 2.
Data Sharing Statement
