# Supplementary material for: Hospital Decision-Making and Adoption of Health-Related Social Needs Programs in US Hospitals
Source: JAMA Netw Open. 2025 Jun 17;8(6):e2516351. doi: 10.1001/jamanetworkopen.2025.16351 (PMC12175011; doi:10.1001/jamanetworkopen.2025.16351)
Supplement: Supplement 1. — eMethods 1. Survey Weights for Survey Nonresponse eMethods 2. Creating Categorical Variables for Management Participation in Health Equity Goals eMethods 3. Multivariable Logistic Regression [file jamanetwopen-e2516351-s001.pdf]

Supplemental Online Content

Zein D, Cronin CE, Puro N, Franz B, McNeill E, Chang JE. Hospital decision-making and adoption of health-related social needs programs in US hospitals. *JAMA Netw Open*. 2025;8(5):e2516351. doi:10.1001/jamanetworkopen.2025.16351

eMethods 1. Survey Weights for Survey Nonresponse
eMethods 2. Creating Categorical Variables for Management Participation in Health Equity Goals
eMethods 3. Multivariable Logistic Regression

This supplemental material has been provided by the authors to give readers additional information about their work.

© 2025 Zein D et al. *JAMA Network Open*.

eMethods 1: Survey Weights for Survey Nonresponse

We compared the characteristics of hospitals that responded and didn't respond to HRSN and health equity goals items using chi-squared test shown in the table below. To adjust for hospital nonresponse, we constructed weights using logistic regression to predict HRSN and health equity goals item response based on hospital size, ownership, religious, hospital location, and safety-net status. Weights were constructed based on the inverse likelihood of item response to generalize to all US acute care hospitals.

	Non-Respondents (N=2,017)	Respondents (N=2,271)	Test
Bed Size			
<50	886 (43.9%)	729 (32.1%)	<0.001
50-199	703 (34.9%)	756 (33.3%)	
200-399	305 (15.1%)	443 (19.5%)	
400+	123 (6.1%)	343 (15.1%)	
Ownership Type			
Nonprofit	953 (47.2%)	1,814 (79.9%)	<0.001
Public	542 (26.9%)	372 (16.4%)	
For-profit	522 (25.9%)	85 (3.7%)	
Religious			
No	1,816 (90.0%)	2,001 (88.1%)	0.044
Yes	201 (10.0%)	270 (11.9%)	
Hospital Location			
Rural	618 (30.6%)	431 (19.0%)	<0.001
Urban	1,399 (69.4%)	1,840 (81.0%)	
Safety-Net Status			
No	1,827 (90.6%)	1,896 (83.5%)	<0.001
Yes	190 (9.4%)	375 (16.5%)	

© 2025 Zein D et al. *JAMA Network Open*.

eMethods 2: Creating Categorical Variables for Management Participation in Health Equity Goals

Below, we include the questions asked in the AHA Survey used to create our key predictor of interest:

Who in your hospital or health care system is accountable for meeting health equity goals? (Check all that apply)

a. CEO

b. Designated Senior Executive (Chief Diversity Office, VP for DEI, etc.)

c. Middle Management

d. Committee or Task Force

e. Division/Department Leaders

f. Employee Resource Group

g. None of the above

Creating our variable: This predictor was measured as a series of binary variables including CEO, senior executive, middle management, committee, division/department leaders, employee resource group and none of the above. We grouped CEO and Designated Senior Executive as senior management and grouped middle management, committee or task force, division/department leaders, employee resource group as middle management. We created a categorical variable identifying whether only senior management (senior only), only other management (others only), both senior and other management (senior + others), or none were selected as a response.

© 2025 Zein D et al. *JAMA Network Open*.

eMethods 3: Multivariable Logistic Regression

Separate multivariable logistic regressions estimated associations between management involvement and each HRSN program adoption while controlling for, hospital characteristics (bed size, ownership, religious, hospital location, and safety net), and county characteristics (percentage of population Black, percentage of population Hispanic, percentage of population Uninsured, ADI).

© 2025 Zein D et al. *JAMA Network Open*.
