# Supplementary material for: Hospital Decision-Making and Adoption of Health-Related Social Needs Programs in US Hospitals
Source: JAMA Netw Open. 2025 Jun 17;8(6):e2516351. doi: 10.1001/jamanetworkopen.2025.16351 (PMC12175011; doi:10.1001/jamanetworkopen.2025.16351)
Supplement: Supplement 2. — Data Sharing Statement [file jamanetwopen-e2516351-s002.pdf]

Data Sharing Statement

Zein. Hospital Decision-Making and Adoption of Health-Related Social Needs Programs in US Hospitals. *JAMA Netw Open*. Published June 17, 2025.
doi:10.1001/jamanetworkopen.2025.16351

Data
Data available: No
